# Over-Expression of Nerve Growth Factor-β in Human Cholangiocarcinoma QBC939 Cells Promote Tumor Progression

**DOI:** 10.1371/journal.pone.0062024

**Published:** 2013-04-24

**Authors:** Xiu-jing Yue, Lei-bo Xu, Man-sheng Zhu, Rui Zhang, Chao Liu

**Affiliations:** Department of Hepato-Pancreato-Biliary Surgery, Sun Yat-Sen Memorial Hospital, Sun Yat-Sen University, Guangzhou, Guangdong Province, China; Institute for Systems Biology, United States of America

## Abstract

**Aims:**

It has been shown that nerve growth factor-β (NGF-β) promoted the initiation and progression of many tumors, and we have previously demonstrated that the expression of NGF-β was associated with tumor stage, nerve infiltration and lymph node metastasis in human hilar cholangiocarcinoma. However, whether NGF-β promotes tumor progression in human cholangiocarcinoma requires further investigation. Therefore, we aimed to determine the effects of NGF-β on the progression of human cholangiocarcinoma.

**Methods:**

Human cholangiocarcinoma QBC939 stable cell lines with over-expressed or silenced NGF-β genes were generated with pEGFP-N1-NGF-β and pGPU6/GFP/Neo-NGF-β-shRNA recombinant plasmids. Cell proliferation assay, colony formation assay, cell cycle analysis, apoptosis assay and tumorigenicity assay were performed to evaluate the role of NGF-β in the progression of human cholangiocarcinoma. In addition, human lymphatic endothelial cells were co-cultured with QBC939 culture supernatants, and the cell proliferation and migration abilities of the lymphatic endothelial cells were evaluated.

**Results:**

Forced expression of NGF-β in QBC939 cell lines promoted proliferation, colony formation and tumorigenicity in these cells and inhibited the apoptosis. However, down-regulation of NGF-β inhibited proliferation, colony formation and tumorigenicity, and increased the apoptotic rate of QBC939 cells. In addition, the NGF-β gain-of-function induced a high expression of vascular endothelial growth factor C and enhanced the proliferation and migration of lymphatic endothelial cells, while NGF-β loss-of-function showed opposite effects.

**Conclusions:**

We concluded that NGF-β promoted tumor progression in human cholangiocarcinoma QBC939 cells. Our results provided a new concept to understand the role of NGF-β in cholangiocarcinoma progression, and might provide important information for the development of new targeted therapies in human cholangiocarcinoma.

## Introduction

Cholangiocarcinoma (CCA) is the second most common primary liver cancer after hepatocellular carcinoma, originating from biliary tract epithelial cells [1]. CCA is characterized by a progressive increase in incidence and prevalence, and is associated with poor prognosis [2]. The treatment of CCA remains a challenge because of the aggressive nature of the disease [3]. Lymphatic dissemination and nerve infiltration are important prognostic factors in CCA. It has been reported that lymphatic node involvement is present in about 50% of the patients undergoing surgery for CCA, which is often associated with a worse outcome [4]. Nerve infiltration has also been shown to reduce the survival rates [5]. However, little is known about the mechanism of tumor initiation, progression, and metastasis formation of CCA.

Nerve growth factor (NGF), a well-known neurotrophin that plays a crucial role in neuronal cell survival and differentiation, is critical for the development and maintenance of nervous system [6]. In fact, there is growing evidence that NGF is able to exert a wide spectrum of effects on many other cells [7] as well as being involved in a wide variety of functions, such as angiogenesis mediation [8] and cancer development promotion [9]. NGF-β is the most important member of the NGF family, which displays the biological activity of NGF. Emerging evidence has shown that NGF-β promoted tumor initiation and progression in many human tumors, such as breast [9], prostate [10], and oral cancers [11]. Furthermore, we have previously demonstrated that the expression of NGF-β was associated with tumor stage, lymph node metastasis and nerve infiltration in human hilar cholangiocarcinoma [5]. However, whether NGF-β could promote tumor progression in human CCA required further investigation.

Therefore, the goal of this study was to evaluate the role of NGF-β in the progression of human CCA. In this study, cell proliferation assay, colony formation assay, cell cycle analysis, apoptosis assay *in vitro* and *in vivo* tumorigenicity assay were performed to evaluate the role of NGF-β in the progression of human CCA. We found that over-expression of NGF-β in the human CCA cell line QBC939 stimulated proliferation, colony formation and tumorigenicity, and inhibited apoptosis of the QBC939 cells. These data suggested that NGF-β promoted tumor progression in human CCA. Our results provided a new concept to understand the role of NGF-β in CCA progression and might provide important information for the development of new targeted therapies in human CCA.

## Materials and Methods

### Reagents and antibodies

The pEGFP-N1 vector was purchased from Clontech Laboratories Inc. (Clontech, CA, USA). The pGPU6/GFP/Neo-NGF-β-shRNA recombinant plasmid was constructed by Shanghai GenePharma Co., Ltd (Shanghai, china). The NGF-β rabbit polyclonal antibody and β-actin mouse monoclonal antibody were purchased from Santa Cruz Biotechnology (Santa Cruz, CA, USA). TurboFectin Transfection Reagent was obtained from Thermo Fisher Scientific Inc. (Waltham, MA, USA). Cell Counting Kit-8 (CCK-8) was purchased from the Dojindo Molecular Technologies (Gaithersburg, MD, USA).

### Cell lines

Human CCA cell line QBC939 was purchased from the Cell Bank of Chinese Academy of Sciences (Shanghai, China) and was cultured in RPMI-1640 (Invitrogen Corp., Carlsbad, CA, USA) supplemented with 10% heat-inactivated fetal bovine serum (FBS; Thermo Fisher Scientific), as recommended by the supplier. Human lymphatic endothelial cells (LECs) and endothelial cell medium were purchased from ScienCell (San Diego, CA, USA).

### Construction of recombinant plasmid and generation of stable cell line

Standard molecular biology techniques were used for construction of the pEGFP-N1-NGF-β recombinant plasmid. The PCR product of human NGF-β fragment was restricted and inserted between Hind III and BamH I restriction sites in the pEGFP-N1-basic vector. The forward and reverse primers (restriction sites are underlined) used to amplify this fragment included 5′- ATTCAAGCTTATGTCCATGTTGTTCTACACTC-3′ and 5′- ATTCGGATCCCGGGCTCTTCTCACAGCCTTCCTG-3′, respectively. Plasmids were transfected into QBC939 cells at the indicated concentrations using TurboFectin Transfection Reagent according to the manufacturer's instructions. Two days after the transfection, we started the stable cell line selection with optimal concentration of G418. The media was changed every 2–3 days and the cells were split when necessary. After 2–4 weeks, all the non-transfected cells were disappeared and isolated colonies began to appear. The NGF-β protein expression was checked in these cells by western blot.

### Cell proliferation and colony formation assay

Cell proliferation assays were conducted using CCK-8 according to the manufacturer's protocol. Briefly, 10 μL of CCK-8 water-soluble formazan dye was added to each well and incubated for 2 h at 37°C in a humidified 5% CO_2_ incubator. The absorbance was measured at 450 nm by an enzyme linked immunosorbent assay (ELISA) microplate reader.

For colony formation assay, 50 cells were seeded into six-well plate and were maintained in RPMI-1640 containing 2% FBS for 10–14 d. Colonies were fixed with 4% paraformaldehyde and were stained with Crystal Violet. The number of cells was counted in each colony and their pictures were captured.

### Cell cycle analysis and apoptosis assay

For cell cycle analysis, QBC939 stable cell lines were cultured for 24 hours. The cells were re-plated and fixed in 70% ethanol overnight at 4°C. After being washed with Dulbecco's Phosphate Buffered Saline (DPBS), the cells were incubated in DPBS containing 20 μg/mL of propidium iodide (PI), 200 μg/mL of RNase A, and 0.1% Triton X-100 at 4°C for 30 min. The stained cells were analyzed for cell cycle distribution by flow cytometry.

Apoptotic rate was detected by Annexin V/PI double staining. Briefly, the cells were washed twice with complete medium and were incubated in 200 μL 1× Binding buffer, 5 μL Annexin V, and 2 μL PI for 15 min at 37°C in the dark. The stained cells were then analyzed by flow cytometry within 1 h.

### Tumorigenicity assays in nude mice

This study was approved by the Institutional Animal Care and Use Committee at Sun Yat-Sen Memorial Hospital, Sun Yat-sen University. Ten million cells of QBC939 stable cell line were suspended in 100 μL DPBS and were subcutaneously injected into female BALB/c athymic nude mice at 5–6 wk of age. Mice were observed daily and were inspected for tumor growth three times a week for at least five weeks. Tumor volume (V) was monitored by measuring the length (L) and width (W) with calipers and was calculated with the formula (L×W^2^) ×0.5.

### Transwell migration assay

The migration ability of LECs was estimated using transwells coated with extracellular matrix gel that was obtained from Corning Inc. (Corning, NY, USA). An aliquot of 1×10^4^ LECs was placed in the upper chamber with 0.1 mL serum-free medium, whereas the lower chamber (24-well plate) was loaded with an aliquot of 5×10^4^ QBC939 cells in 0.5 mL of RPMI-1640 containing 10% FBS. After 48 h of incubation, LECs that were still on the upper side of the filters were mechanically removed with cotton swabs. Cells that migrated to the lower side were fixed with 4% paraformaldehyde and were counterstained with 0.1% crystal violet. The cells that had migrated into the lower chamber were observed and counted under a light microscope. Finally, the number of the migrating cells was calculated.

### Western blot analysis

Protein lysates from the cells in culture were subjected to sodium dodecyl sulfate-polyacrylamide gel electrophoresis (SDS-PAGE) and were detected with primary antibodies recognizing NGF-β (1∶1000) and β-actin (1∶5000), respectively.

### ELISA

VEGF-C concentrations in QBC939 culture supernatants were assessed by ELISA using the VEGF-C (human) ELISA Kit (RayBiotech, Norcross, GA, USA) according to the manufacturer's instructions.

### Statistical analysis

All experiments were performed at least in triplicate. Statistical analysis was conducted with the SPSS software package (version 16.0; SPSS, Chicago, IL, USA). Quantitative data were presented as mean ± standard deviation (SD) unless otherwise indicated, and the Student's *t*-test was used for evaluating the statistical significance. For all tests, a P value of less than 0.05 was considered to indicate statistical significance and was indicated by asterisks in the figures.

## Results

### Generation and identification of QBC939 stable cell lines with over-expressed or silenced NGF-β

**Figure 1:**
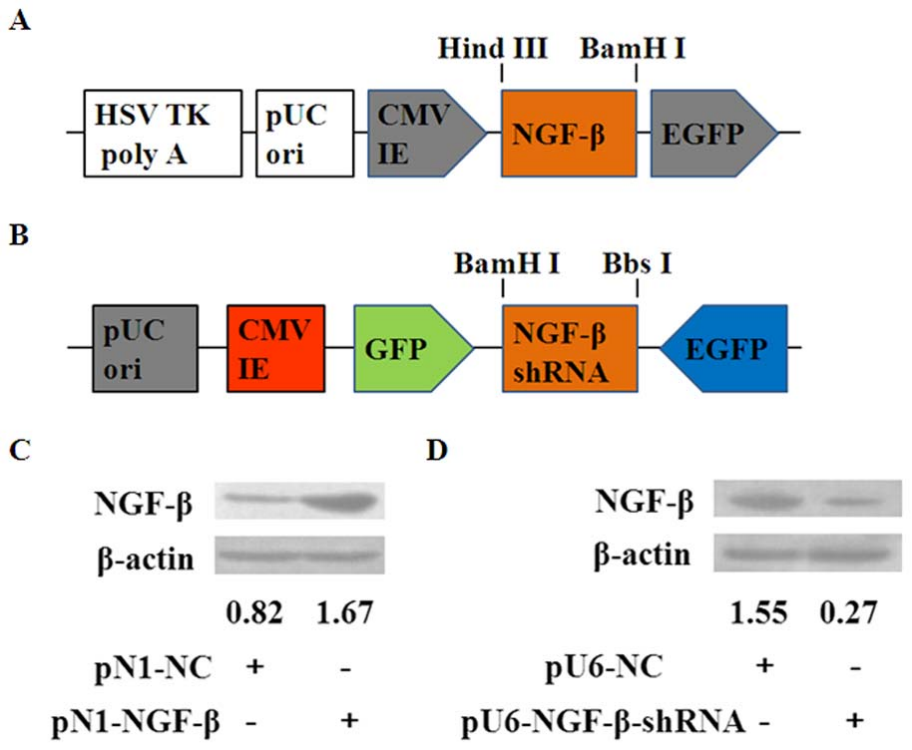
Generation and identification of QBC939 stable cell lines with over-expressed or silenced NGF-β. (A) The PCR product of human NGF-β fragment was restricted and inserted between Hind III and BamH I restriction sites in the pN1-basic vector. (B) The PCR product of human NGF-β shRNA fragment was restricted and inserted between BamH I and Bbs I restriction sites in the pU6-basic vector. (C) Western blot analysis showed that NGF-β protein level was significantly up-regulated in the cells transfected with pN1-NGF-β recombinant plasmid as compared to the negative control. (D) A significant down-regulation of NGF-β protein in QBC939 cells caused by pU6-NGF-β-shRNA transfection.

To obtain the QBC939 stable cell lines with over-expressed or silenced NGF-β, the pEGFP-N1-NGF-β (pN1-NGF-β; over-expressed) and pGPU6/GFP/Neo-NGF-β-shRNA (pU6-NGF-β-shRNA; silenced) recombinant plasmids were transfected into the QBC939 cells, respectively. The QBC939 cells were also stably transfected with the pEGFP-N1 and pGPU6/GFP/Neo basic vector to serve as negative control (pN1-NC and pU6-NC). The QBC939 stable cell lines were selected with G418 Sulfate and Western blot was performed to investigate the expressions of NGF-β protein in these cell lines. Our data showed that high expression of NGF-β protein was detected in cells that were transfected with pN1-NGF-β recombinant plasmid as compared to cells that were transfected with pN1-NC (Figure 1A and C). Cells that were transfected with pU6-NGF-β-shRNA exhibited 17.4% of the expression level that was displayed by the corresponding negative control (pU6-NC; Figure 1B and D).

### NGF-β promoted tumor growth *in vitro* and tumorigenicity *in vivo*

**Figure 2:**
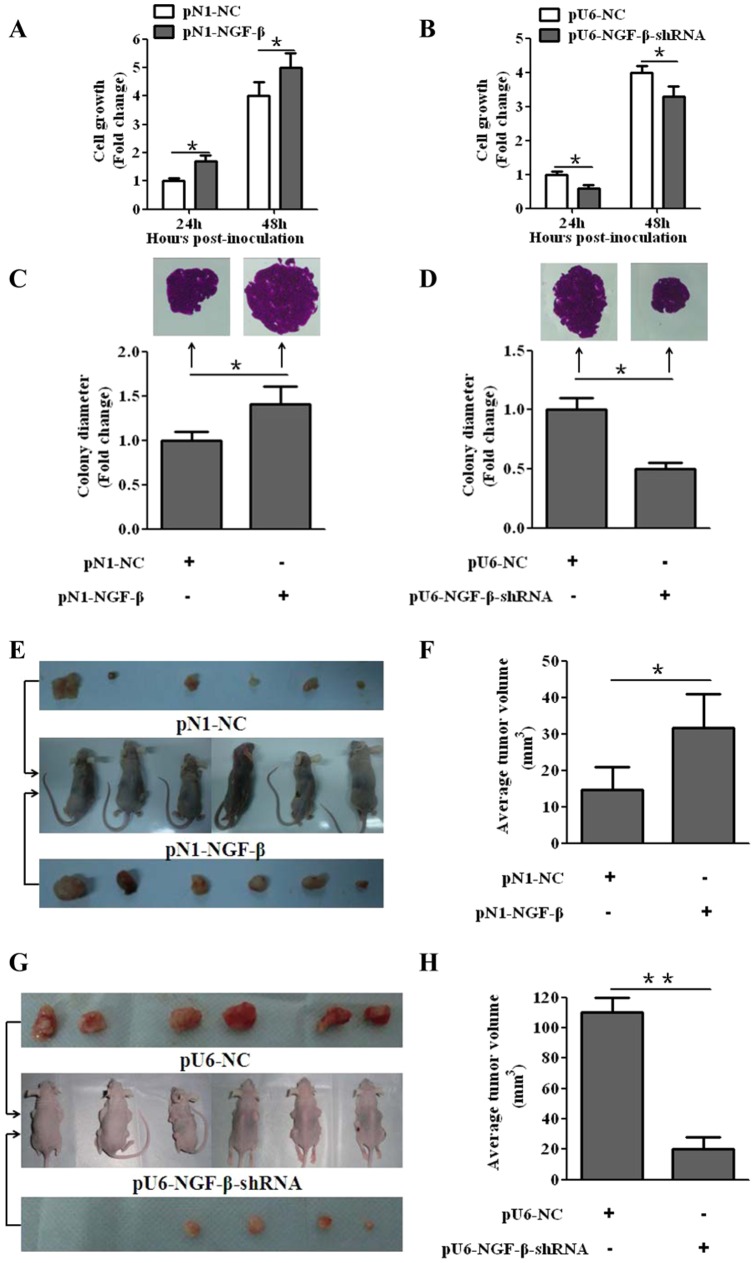
NGF-β promoted tumor growth *in vitro* and tumorigenicity *in vivo*. (A) NGF-β promoted tumor growth of QBC939 cells. (B) Knockdown of NGF-β by NGF-β-shRNA repressed the proliferation of the QBC939 cells. (C) NGF-β enhanced colony formation capability of the QBC939 cells. (D) NGF-β silencing by NGF-β-shRNA inhibited the colony formation capability of QBC939 cells. (E) The photographs of all the nude mice and all the dissected tumors from the QBC939 stable cell lines over-expressing NGF-β at the end of the experiments (5 wk). (F) Average tumor volumes from the QBC939 stable cell lines over-expressing NGF-β (n = 6). (G) The photographs of all the nude mice and all the dissected tumors from QBC939 stable cell lines silenced for NGF-β expression at the end of the experiments (6 wk). (H) Average tumor volumes from QBC939 stable cell lines silenced for NGF-β function (n = 6). *P<0.05, **P<0.001.

To explore the possible biological significance of NGF-β in CCA tumorigenesis, proliferation assay and colony formation assay was performed as an initial step. The results showed that NGF-β dramatically promoted tumor growth of QBC939 cells and enhanced their colony formation capability *in vitro* (P<0.05; Figure 2A and C). Consistent with the above results, the pU6-NGF-β-shRNA transfection caused tumor growth inhibition and decreased the size of the colonies (P<0.05; Figure 2B and D). This result was further confirmed by tumorigenicity assays in nude mice. The volumes of tumors derived from pN1-NGF-β cells were much larger than that of the tumors originating from pN1-NC cells (P<0.05; Figure 2E and F). Accordingly, the volumes of tumors derived from pU6-NGF-β-shRNA cells were smaller than that of the tumors originating from pU6-NC cells (P<0.05; Figure 2G and H).

### NGF-β promoted cell cycle progression and decreased apoptotic rates of QBC939 cells

**Figure 3:**
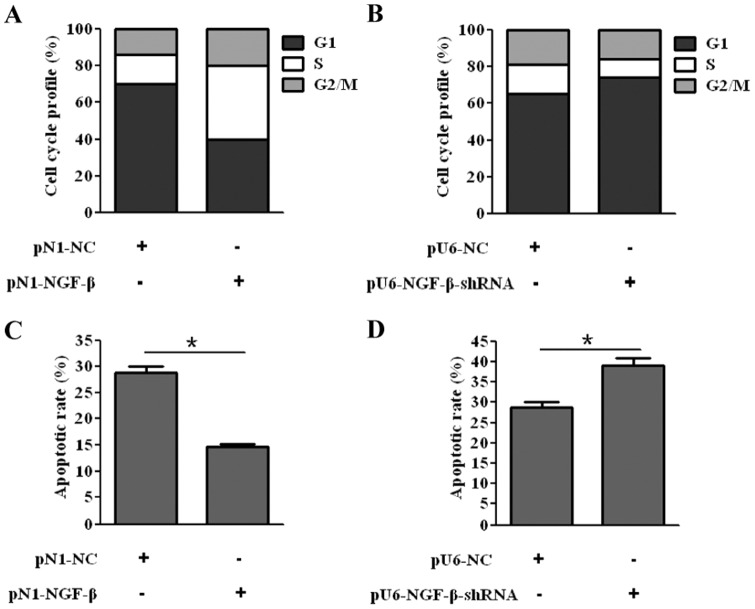
NGF-β promoted cell cycle progression and decreased apoptotic rates of QBC939 cells. Representative histograms are displayed. (A) Acceleration of the G1/S phase transition by NGF-β. (B) Retardation of the G1/S phase transition by NGF-β-shRNA. (C) NGF-β inhibited apoptosis of QBC939 cells. (D) NGF-β silencing promoted apoptosis of QBC939 cells.

We further assessed the role of NGF-β in cell cycle progression and apoptosis of QBC939 cells. The results showed that the percentages of the cells in the S phase among the cells transfected with pN1-NC and pN1-NGF-β were 15.64±0.69% and 40.19±1.67%, respectively (Figure 3A). The corresponding percentages of the cells transfected with pU6-NC and pU6-NGF-β-shRNA were 15.95±0.72% and 10.07±1.01%, respectively (Figure 3B). These data indicated that NGF-β could speed up the G1/S phase transition and NGF-β-shRNA could slow down the G1/S phase transition, respectively (Figure S1). In addition, the apoptotic rates were significantly decreased by NGF-β transfection (Figure 3C), while significantly increased by NGF-β-shRNA transfection (Figure 3D). Therefore, NGF-β could also decrease the apoptotic rates of QBC939 cells (Figure S2).

### NGF-β over-expression enhanced proliferation and migration of LECs through up-regulation of VEGF-C

**Figure 4:**
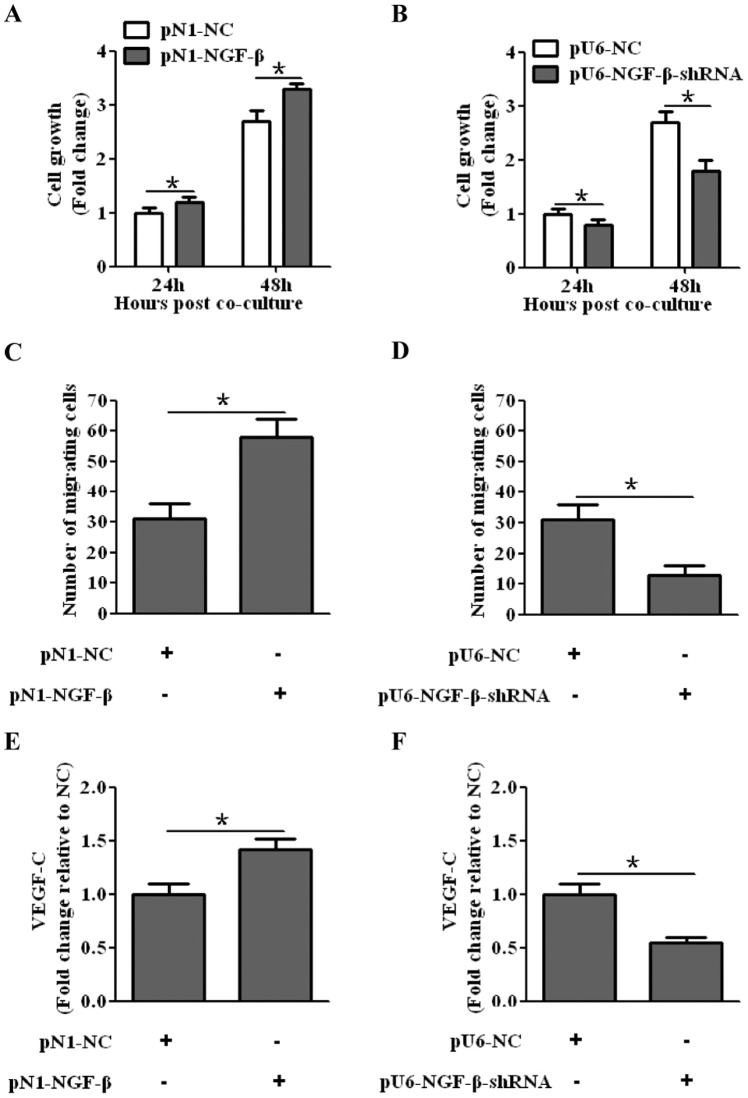
NGF-β might facilitate lymphatic metastasis through up-regulation of VEGF-C. (A) The LEC that was co-cultured with QBC939 stable cell lines transfected with pN1-NGF-β showed a higher proliferative ability as compared to the corresponding negative control. (B) Co-culture of QBC939 stable cell lines with silenced NGF-β reduced the proliferation of LECs. (C) The LEC that was co-cultured with QBC939 stable cell lines transfected with pN1-NGF-β showed higher migration ability. (D) Co-culture of QBC939 stable cell lines with silenced NGF-β reduced the migration of LECs. (E) NGF-β up-regulated the expression of VEGF-C. (F) The expression of VEGF-C was down-regulated in the culture supernatant of the QBC939 stable cell line with silenced NGF-β.

We have previously demonstrated that the expression of NGF-β was associated with lymph node metastasis in human hilar cholangiocarcinoma. However, whether NGF-β could facilitate lymphatic metastasis and its precise molecular mechanism remained unclear. In this study, the effects of NGF-β on the proliferation and migration activity of LECs were evaluated by cell proliferation and transwell assay. Culture supernatant of the QBC939 stable cell lines were collected after 48 hours of incubation and human LECs were cultured in it. Meanwhile, the concentration of VEGF-C in the culture supernatant was assessed by ELISA. We found that the LEC that was co-cultured with QBC939 stable cell lines transfected with pN1-NGF-β showed a higher proliferative and migration abilities as compared to the corresponding negative control (Figure 4A and C). In contrast, co-culture with QBC939 stable cell lines with silenced NGF-β reduced the proliferation and migration of LECs (Figure 4B and D; Figure S3). In addition, the expression of VEGF-C was markedly increased by NGF-β gain-of-function (Figure 4E), while NGF-β loss-of-function by NGF-β-shRNA caused a significant down-regulation of VEGF-C (Figure 4F). These data suggested that NGF-β might facilitate the lymphatic metastasis in human CCA through up-regulation of VEGF-C.

## Discussion

NGF is the first member of the neurotrophin family that was discovered and is widely expressed in non-neuronal tissues, such as heart [12], liver [13], lung and skin [14]. High levels of NGF have been detected in many different tumor types and can be targeted to inhibit the tumor cell proliferation, angiogenesis, and metastasis [9]-[11]. Gigliozzi et al. [13] also reported that NGF played a role in modulating the proliferation of cholangiocytes. However, little is known about the expression level and the effects of NGF in human CCA. Our previous studies showed that NGF-β over-expression was observed in most of human hilar cholangiocarcinoma tissues, which was highly correlated with tumor stage, lymph node metastasis and nerve infiltration [5]. However, whether NGF-β could promote tumor initiation, progression and metastasis in human CCA and its molecular mechanisms require further investigation. In this study, we showed that NGF-β promoted tumor proliferation and colony formation *in vitro*, and enhanced the tumorigenicity of human CCA cell line QBC939 *in vivo*. We also observed that NGF-β promoted cell cycle progression and decreased apoptotic rates of QBC939 cells. In addition, NGF-β loss-of-function by NGF-β shRNA showed opposite effects. All these findings emphasized a fundamental role of NGF-β in the progression of human CCA.

Lymphatic vessel invasion and lymph node metastasis are major negative prognostic factors for CCA [15], [16]. However, the mechanism by which tumor cells infiltrate the lymphatic system is still a subject of debate. Recently, emerging evidence has shown that lymph node metastasis might be facilitated by tumor-stimulated lymphangiogenesis, the formation of new lymphatic vessels from pre-existing lymphatic vessels [17]. Similar to angiogenesis, lymphangiogenesis is also an important process that contributes to progression and metastasis of many kinds of malignant neoplasms, including CCA. Tumors can produce a range of pro-lymphangiogenesis inducers, such as VEGF-C, that directly or indirectly stimulate lymphangiogenesis and lymphatic metastasis [18]. To validate whether NGF-β could facilitate lymphatic metastasis, human LECs were co-cultured with QBC939 culture supernatants, and the cell proliferation and migration abilities of the LECs were evaluated. We found that LEC that was co-cultured with NGF-β high-expressing QBC939 cells showed a higher proliferation and migration abilities as compared to that of the corresponding control. In contrast, co-culture with NGF-β gene-silenced QBC939 cells reduced the proliferation and migration abilities of LECs. This was consistent with our previous results, which showed that NGF-β over-expression was highly correlated with lymph node metastasis [5]. Taken together, these results indicated that NGF-β might facilitate lymphangiogenesis and could potentially serve as a pro-lymphangiogenesis inducer in human CCA.

It is generally accepted that VEGF-C is the most important lymphangiogenic factor that promotes lymphangiogenesis through autocrine and paracrine process [19], [20]. Recently, it was reported that VEGF-C expression was significantly correlated with lymph node metastasis and could be considered as an independent and important prognostic factor in intrahepatic cholangiocarcinoma patients [21]. Aishima et al. [22] also reported that VEGF-C expression was significantly correlated with lymphatic invasion and acted as an independent prognostic factor in intrahepatic cholangiocarcinoma. In hilar cholangiocarcinoma, our previous results showed that VEGF-C over-expression was observed in 46.4% of the tumor samples and was highly correlated with lymphangiogenesis and lymph node metastasis. Interestingly, NGF-β over-expression was highly correlated with VEGF-C over-expression [5]. These results prompted us to investigate whether NGF-β facilitated lymphatic metastasis though up-regulation of VEGF-C. Therefore, culture supernatants of QBC939 cells with highly-expressed or silenced NGF-β gene were collected after 48 hours of incubation. The VEGF-C concentrations were measured in the culture supernatants using ELISA. Our analysis revealed that the expression of VEGF-C was markedly increased by NGF-β gain-of-function, and NGF-β loss-of-function by NGF-β-shRNA caused a significant down-regulation of VEGF-C. Taken together, these results indicated that NGF-β modulated the expression of VEGF-C. However, whether NGF-β affects lymph node metastasis directly via NGF-β-induced lymphangiogenesis or indirectly via the induction of classical “lymphangiogenic factors” such as VEGF-C, remains to be clarified.

In summary, our data suggested a crucial role of NGF-β in the progression and metastasis of cancer, and might be potentially helpful for the development of new targeted therapies in human CCA. However, it is necessary to evaluate the pro-tumor effects of NGF-β in more than two cell lines to verify it is not a cell line-specific phenomenon. Furthermore, induction of NGF-β gene silencing by a second shRNA would further strengthen these results. The potential pro-lymphangiogenesis effect of NGF-β and the underlying mechanisms are still largely unknown and more extensive investigations are required.

## Supporting Information

Figure S1 **NGF-β promoted cell cycle progression of QBC939 cells.** The QBC939 stable cell lines were cultured for 24 hours after being re-plated and the distribution of the cells in cell-cycle phases was assessed by flow cytometry using propidium iodide staining.
(TIF)

Figure S2 **NGF-β decreased the apoptotic rates of QBC939 cells.** The QBC939 stable cell lines were cultured for 24 hours after being re-plated and apoptotic rates were detected by flow cytometry using Annexin V/PI double staining. The lower left quadrants of the density plots indicate viable cells staining negative for FITC Annexin V and PI. The subpopulation of cells staining PI negative and FITC Annexin V positive is in early apoptosis (lower right quadrant). The subpopulation of cells staining PI positive and FITC Annexin V negative is dead cells (upper left quadrant), while those that stain with both FITC Annexin V and PI (upper right quadrant) are in late apoptosis. Apoptotic rate was determined on the basis of Annexin V+/PI+ and Annexin V+/PI− fractions.
(TIF)

Figure S3 **NGF-β up-regulated the migration ability of LECs.** The migration ability of LECs was estimated using transwells coated with extracellular matrix gel. The cells that had migrated into the lower chamber were observed and counted under a light microscope. LECs co-cultured with QBC939 stable cell line that transfected with pN1-NGF-β showed higher migration ability, while co-culture with QBC939 stable cell lines that silenced NGF-β reduced the migration ability of LECs.
(TIF)
