# Supplementary figures and images for: Over-Expression of Nerve Growth Factor-β in Human Cholangiocarcinoma QBC939 Cells Promote Tumor Progression
Source: PLoS One. 2013 Apr 24;8(4):e62024. doi: 10.1371/journal.pone.0062024 (PMC3634741; doi:10.1371/journal.pone.0062024)

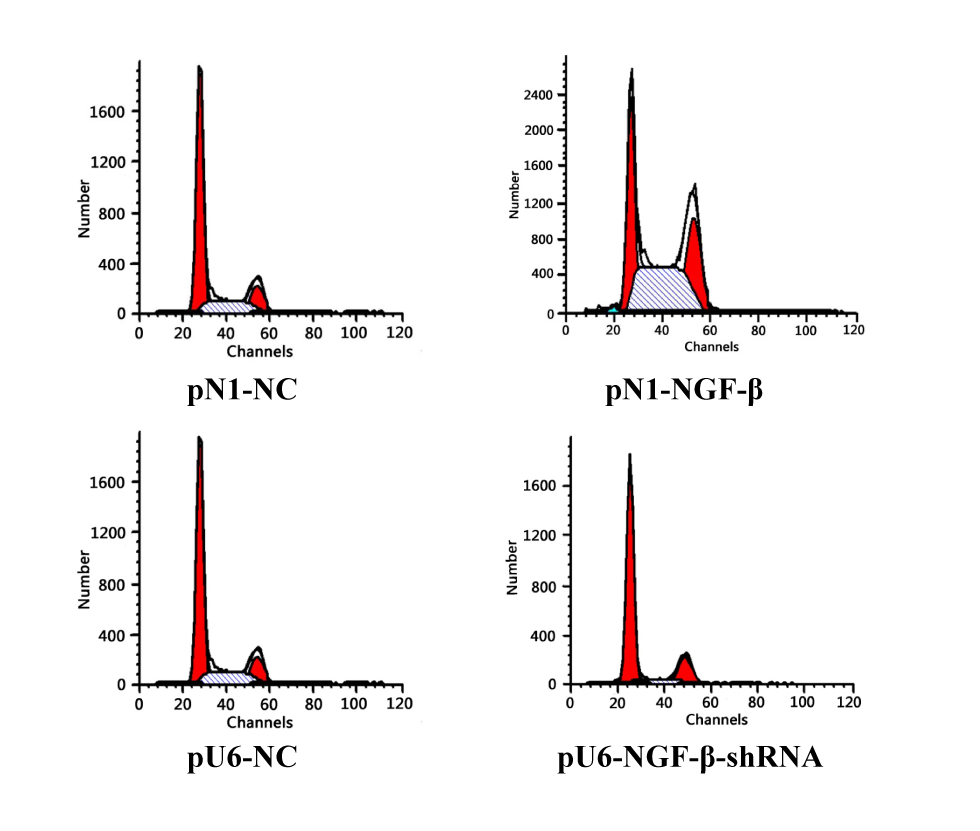

Supplement: Figure S1 — NGF-β promoted cell cycle progression of QBC939 cells. The QBC939 stable cell lines were cultured for 24 hours after being re-plated and the distribution of the cells in cell-cycle phases was assessed by flow cytometry using propidium iodide staining. (TIF) [file pone.0062024.s001.tif]

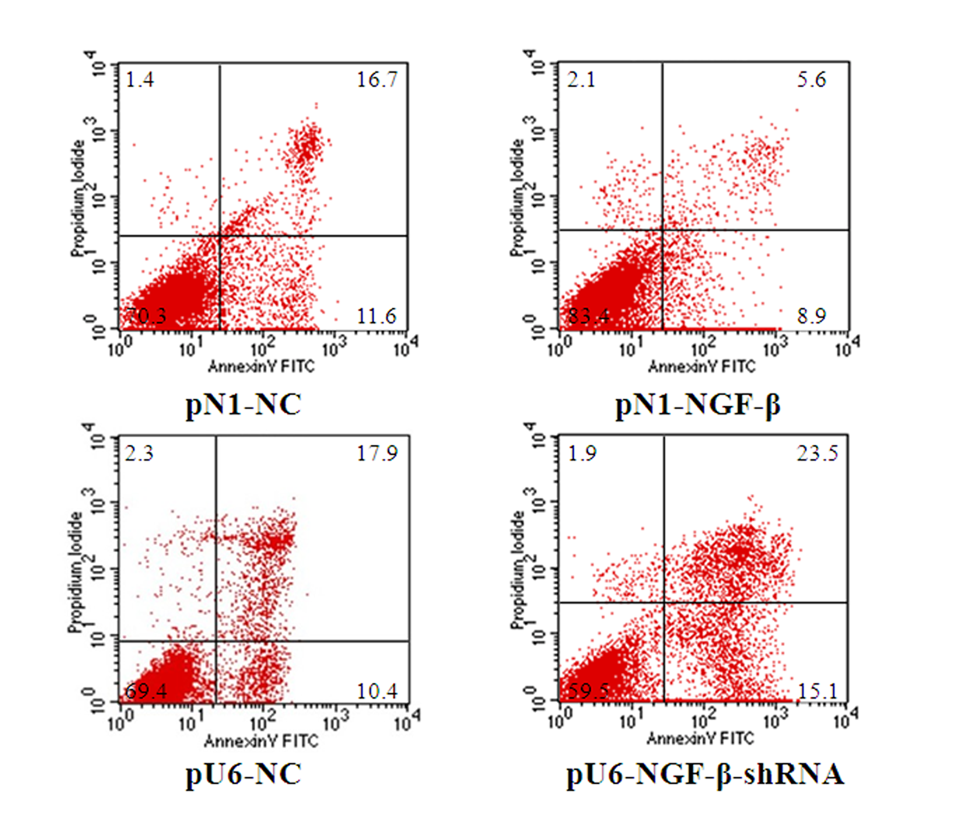

Supplement: Figure S2 — NGF-β decreased the apoptotic rates of QBC939 cells. The QBC939 stable cell lines were cultured for 24 hours after being re-plated and apoptotic rates were detected by flow cytometry using Annexin V/PI double staining. The lower left quadrants of the density plots indicate viable cells staining negative for FITC Annexin V and PI. The subpopulation of cells staining PI negative and FITC Annexin V positive is in early apoptosis (lower right quadrant). The subpopulation of cells staining PI positive and FITC Annexin V negative is dead cells (upper left quadrant), while those that stain with both FITC Annexin V and PI (upper right quadrant) are in late apoptosis. Apoptotic rate was determined on the basis of Annexin V+/PI+ and Annexin V+/PI− fractions. (TIF) [file pone.0062024.s002.tif]

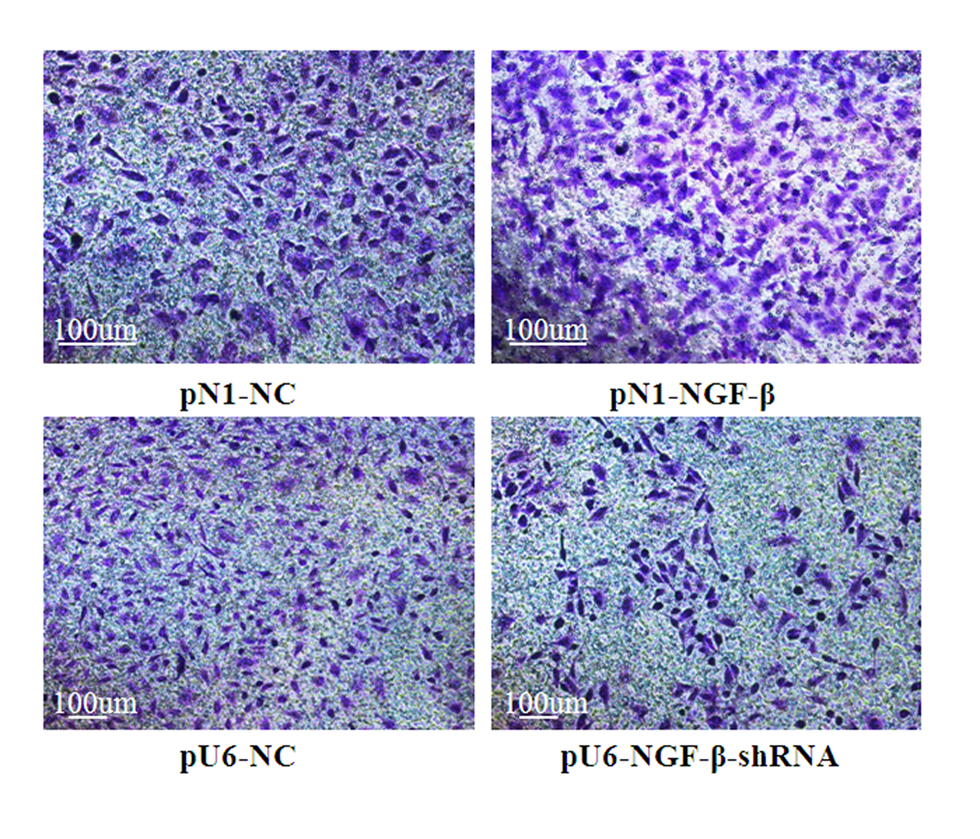

Supplement: Figure S3 — NGF-β up-regulated the migration ability of LECs. The migration ability of LECs was estimated using transwells coated with extracellular matrix gel. The cells that had migrated into the lower chamber were observed and counted under a light microscope. LECs co-cultured with QBC939 stable cell line that transfected with pN1-NGF-β showed higher migration ability, while co-culture with QBC939 stable cell lines that silenced NGF-β reduced the migration ability of LECs. (TIF) [file pone.0062024.s003.tif]
